# On the Treatment of Cancer by the Lactate of Iron, Taken by the Mouth and Injected into the Veins

**Published:** 1852-08

**Authors:** Daniel Brainard

**Affiliations:** Chicago


### SELECTIONS.

From the American Journal of Medical Sciences.

*On the Treatment of Cancer by the Lactate of Iron, taken by the mouth and injected into the veins.* By DANIEL BRAINARD, M.D.

ABOUT two years since, I communicated to Prof. Mussey, chairman of the Committee on Surgery of the American Medical Association, some reasons which I had for supposing that the lactate of iron was possessed of more influence over cancer than any other medicine yet known.

I have, since that time, had occasion to prescribe it often, with results which, while they confirm the views expressed in regard to its efficiency in checking it, have not shown that it was capable of entirely curing it. This result was to me neither surprising nor discouraging, as I have already formed and expressed the opinion, that to effect a cure, "the whole of the solids and fluids of the body must be brought under its influence." That this is not effected by the simple introduction of medicines into the stomach, is sufficiently obvious, and indeed to be expected, since the medicine, used in that way, is subjected to the action of the same nutrition and absorption under the influence of which the disease has originated. It is necessary to go behind this; and one of the means of doing so is by injecting it into the veins. It is only recently that I have had an opportunity of putting this method to the test of practice.

CASE.—Dec. 14, 1851. W. H. Plum, æt. 56 years, Englishman, applied to me on account of a tumor of the left orbit.

He gave the following history of his disease:

"About twenty-five years ago he had a disease of that eye, called by his Physician cataract, which entirely destroyed the vision, but for which no operation was performed. About five years ago he received an injury of that eye, from a stick striking against it, which was slight, and gave but little pain. About seven months after this blow, he noticed a tumor, no larger than a pea, at the inner canthus, 'sending off roots into the eye-ball.' At this time the tumor and eye-ball were removed together by Prof. Smith of Baltimore. The wound cicatrized well.

He remained in pretty good health about four years, when a tumor made its appearance at the lower and inner part of the orbit, which in eight months attained the size of a large hickory nut. It was then operated upon again, but at the end of about six weeks recommenced to grow, and at the time of this examination was of the size of an orange, filling up the whole of the orbit and projecting in front of it. Its surface was nodulated, elastic, pulsating, ulcerated to a great extent, and from this point there oozed a bloody serous fluid. He was thin, but not sallow; and his health was not very much impaired. He complained, however, of acute lancinating pains through the orbit and head.

16th. Extirpation was performed in presence of the hospital class. It was found so firmly attached to the lower part of the orbit, that it was necessary to remove the periosteum with it, and at the back part it could not all be removed. There remained a muscular mass, which bled profusely, and which was so soft as to break under the forceps or tenaculum. After several ineffectual attempts to apply a ligature, the actual cautery was resorted to and succeeded. The wound was dressed with lint. No inflammation followed. There was a copious discharge of red serum for a day or two, which gradually became yellow, and afterwards changed to pus. He was put, from the day of the operation, on the use of lactate of iron gr. v, three times a day in solution.

31st. Injected into his veins f ʒ j of the following solution:

Ferri lactis, gr. viij
Aq. dist., ℥j

Jan. 3, 1852. Injected ʒij of the same solution.

6th. f ʒ iij thrown in.

14th. ʒ ijss injected.

22d. ʒij.

26th. ʒ ij.

28th. ʒ ij.

Feb. 3d. ʒ ij injected. 9th. ʒ ijss.

During the whole of this time the wound cicatrized rapidly. At first luxuriant granulations sprang from the surface, which were repressed by the application of nit. silver. Lancinating pains continued for some time, but gradually diminished, and at length subsided.

In six weeks from the operation the cicatrization was nearly complete. In eight weeks he returned home perfectly well.

The question, whether the diseased mass was a cancer, I do not hesitate to decide in the affirmative. Its history and appearance sufficiently indicate this; its interior perfectly resembled the brain of an infant in a vascular state, and under the microscope it exhibited the most perfectly formed cancer cells. Dr. Johnson, resident physician, fully coincided in this point.

Whether it would have cicatrized without the use of the lactate of iron, cannot be determined with the same degree of certainty. Taking into consideration the return, when last extirpated, with the fact that it was afterwards impossible to remove the whole of it, I think the probability of obtaining cicatrization by ordinary means was slight. I should not, however, have thought of performing, or attempting extirpation, but that the patient, who is intelligent and trusting, expressed his desire to be submitted to the treatment, when it was explained to him.

I am aware that many surgeons, under the influence of preconceived opinions, may regard such treatment as hazardous. I had fully convinced myself that such was not the case. I have repeatedly thrown gr. x lactate of iron, imperfectly dissolved in an ounce of water, into the veins of a small dog, without producing in any case peculiarly bad results.

It will be seen that gr. iij was the largest quantity thrown in at a single time. It was passed in gradually and cold, and as soon as sensible effects were produced, it was stopped. The effect noticed was a flush of the face, a fulness of the veins of the head, and a tendency to sneeze, which all passed over in a few seconds. The circulation otherwise was unaffected. If the case had not progressed favorably, and it had seemed advisable to change the nutrition more profoundly, I would have had the solution warmed and put it in slowly until its effects were perceptible; then allowing it to pass over, have repeated it as far as appeared safe.

Up to the time of his departure, the injection had been performed nine times, and grs. xix in the aggregate injected. When the activity of the salt is considered, it will be conceded that such a quantity is capable of having an effect on the system, by being thrown into the blood.

In addition to that, he has during this period of eight weeks, taken ℨ xix of the lactate by the mouth; to what extent this may have been absorbed and carried into the circulation, or what changes it may have undergone, it is impossible to determine.

In case of a cancerous disease seated upon an extremity, I should, in addition to the two methods of administration resorted to in this instance, infiltrate the whole of the diseased and the healthy tissue about it with a weak solution of the medicine. This can readily be effected by putting a ligature, moderately tight, about the member, until it becomes œdematous, when, by the aid of frictions, the infiltration and maceration may be effected.

I had omitted to mention, that all the injections were made into the veins at the elbow.

In submitting this case to the profession, I am far from claiming for it any merit which it does not possess, or drawing inferences which no single case could warrant. It is offered as an evidence of the practicability and safety of maceration through the medium of the blood, systematically pursued with active substances, and to invite attention to other means of treating this inveterate disease than those which hitherto have been admitted by consent to be unsuccessful.

Chicago, February 21, 1852.

